# More Than (Single) Text Comprehension? – On University Students’ Understanding of Multiple Documents

**DOI:** 10.3389/fpsyg.2020.562450

**Published:** 2020-10-23

**Authors:** Nina Mahlow, Carolin Hahnel, Ulf Kroehne, Cordula Artelt, Frank Goldhammer, Cornelia Schoor

**Affiliations:** ^1^Leibniz Institute for Educational Trajectories (LIfBi), Bamberg, Germany; ^2^DIPF Leibniz Institute for Research and Information in Education, Frankfurt am Main, Germany; ^3^Centre for International Student Assessment (ZIB), Frankfurt am Main, Germany; ^4^Department of Longitudinal Educational Research, University of Bamberg, Bamberg, Germany; ^5^Department of Educational Research, University of Bamberg, Bamberg, Germany

**Keywords:** multiple document comprehension, single text comprehension, university students, reading comprehension, assessment

## Abstract

The digital revolution has made a multitude of text documents from highly diverse perspectives on almost any topic easily available. Accordingly, the ability to integrate and evaluate information from different sources, known as multiple document comprehension, has become increasingly important. Because multiple document comprehension requires the integration of content and source information across texts, it is assumed to exceed the demands of single text comprehension due to the inclusion of two additional mental representations: the integrated situation model and the intertext model. To date, there is little empirical evidence on commonalities and differences between single text and multiple document comprehension. Although the relationships between single text and multiple document comprehension can be well distinguished conceptually, there is a lack of empirical studies supporting these assumptions. Therefore, we investigated the dimensional structure of single text and multiple document comprehension with similar test setups. We examined commonalities and differences between the two forms of text comprehension in terms of their relations to final school exam grades, level of university studies and university performance. Using a sample of *n* = 501 students from two German universities, we jointly modeled single text and multiple document comprehension and applied a series of regression models. Concerning the relationship between single text and multiple document comprehension, confirmatory dimensionality analyses revealed the best fit for a model with two separate factors (latent correlation: 0.84) compared to a two-dimensional model with cross-loadings and fixed covariance between the latent factors and a model with a general factor. Accordingly, the results indicate that single text and multiple document comprehension are separable yet correlated constructs. Furthermore, we found that final school exam grades, level of university studies and prior university performance statistically significant predicted both single text and multiple document comprehension and that expected future university performance was predicted by multiple document comprehension. There were also statistically significant relationships between multiple document comprehension and these variables when single text comprehension was taken into account. The results imply that multiple document comprehension is a construct that is closely related to single text comprehension yet empirically differs from it.

## Introduction

Reading is a core competence for societal and other forms of participation (OECD, 2010). It is assumed to be necessary for knowledge acquisition and skills development. However, reading *per se* as well as the demands readers need to meet have changed a lot as a result of the digital revolution. The ubiquity of the internet allows people to retrieve information and generate knowledge anytime and everywhere. This has led not only to changes in the modality of reading sources from paper-based to computer-based (e.g., Singer and Alexander, 2017; Kroehne et al., 2019), but increasingly requires readers to be able to integrate and evaluate information from different sources (List and Alexander, 2017a) due to the accessibility and multitude of available information. This competence, known as multiple document comprehension (MDC; e.g., Bråten and Strømsø, 2010a), entails the successful understanding, representation and integration of information from texts on the same subject matter stemming from different sources (also referred to as multiple documents).

Integrating and evaluating text information is especially relevant for university students, who need to become familiar with different topics and must be able to autonomously find information in order to study for an exam, give a presentation or review available literature for a term paper. In the course of such tasks, they might encounter multiple documents that provide redundant, complementary or even conflicting information (Bråten et al., 2014). Students have to determine the similarities and differences between texts in order to establish a coherent representation of who said what. There is evidence that a large number of students have problems with the demands of processing more than a single text (for an overview see Britt and Rouet, 2012). However, there are studies indicating that MDC can be improved through interventions (Britt and Aglinskas, 2002) and that it increases over the course of students’ university studies (Schoor et al., 2020b; von der Mühlen et al., 2016).

Until the mid-1990s, models of reading comprehension focused on single text comprehension (STC; e.g., Kintsch, 1988; Trabasso et al., 1989; Graesser et al., 1994; Zwaan et al., 1995), leading to the development of reading comprehension tests based on the extraction of meaning from single texts with a single source. This changed in the late 1990s, when Perfetti et al. (1999) published the documents model framework. This framework addressed the expanded demands of MDC compared to STC by adding additional mental representations. These additional representations are referred to as the integrated situation model (integration of the content of multiple documents) and the intertext model (integration of source information from multiple documents, e.g., the author or publishing date), both of which are part of the documents model (Perfetti et al., 1999; Britt and Rouet, 2012). Since then, numerous researchers have drawn upon this theoretical foundation to build various models that shed light on the different conditions and mechanisms involved in building a documents model. These models concern the interaction between person, task and text, although they focus on different elements (D-ISC model by Braasch and Bråten, 2017; CAEM by List and Alexander, 2017b; two-step validation model by Richter and Maier, 2017; RESOLV model by Rouet et al., 2017; content-source integration model by Stadtler and Bromme, 2014).

Despite these theoretical efforts, the question regarding particular requirements of MDC – and therefore the structure of the relationship between single text and multiple document comprehension – remains insufficiently clarified (Stadtler, 2017; Strømsø, 2017). Since the dimensionality of STC and MDC has not yet been examined, the present study addresses this research gap. Specifically, this study applies a newly developed test measuring MDC (Schoor et al., 2020a, b). The test covers all facets of mental representations within MDC; it thus includes not only the integrated situation model component, as it has often been addressed in former studies using expressive and receptive tasks (for an overview see Primor and Katzir, 2018), but also the intertext model and documents model components. To measure STC, a standardized and approved instrument from the National Educational Panel Study that taps important cognitive requirements for reading was used (Gehrer et al., 2013). Both MDC and STC are abilities that students should learn in school before entering university, but it can be expected that they further develop during students’ university studies (e.g., Schoor et al., 2020b; von der Mühlen et al., 2016, for MDC). Due to our focus on MDC, we also examined the relation between MDC test scores and students’ level of university studies, final school exam grades and university performance. Furthermore, we examined these relations when including STC in the models in order to investigate whether this provides additional insights into the relationship between MDC and STC.

## Theoretical Background

### Single Text Comprehension

Single text comprehension (also often referred to as text or reading comprehension) is the result of a process of extracting meaning from text and establishing a coherent mental representation of the text content. It comprises several cognitive component skills at the word, sentence, and text level (e.g., Perfetti et al., 2005) which also differentiate skilled and poor readers. Reading comprehension is required for literally all higher-level cognitive activities, such as learning, logical thinking, problem solving and decision making (Kintsch, 1988).

Since there is a long research tradition in the field of reading comprehension, different comprehension models have been developed (for an overview of seven prominent models see McNamara and Magliano, 2009). Some have focused on basic and general comprehension processes and verbal efficiency (Perfetti, 1985; Kintsch, 1988; Gernsbacher, 1991; van den Broek et al., 1999), others focus primarily on inference processes and on retrieving prior knowledge (e.g., Trabasso et al., 1989; Graesser et al., 1994; Zwaan et al., 1995). In his seminal paper, Kintsch (1988), suggested that readers construct three layers of representations. (1) The *surface code* or surface level is created through decoding processes of the verbatim text in order to construct a representation of the text string (lexical and syntactical structure). (2) The *textbase level* is the first level of meaning, in which the explicit content of the text is represented by the reader. (3) Deep meaning is established through the construction of a *situation model*. This represents the interpretation level, since prior knowledge and inferences are used here to build an elaborate and coherent interpretation of the information provided in the text.

Taken together, STC involves several cognitive activities and strategies, such as establishing local and global coherence relations, drawing knowledge-based inferences (e.g., Graesser et al., 1994; Oakhill et al., 2003), monitoring the plausibility of the text (Isberner and Richter, 2014) and monitoring the comprehension process itself (Cain, 2009).

When assessing overall reading comprehension abilities, test developers often adopt a result-oriented perspective condensing internal structures and processes. At the same time care has to be taken to ensure that the demands implemented in a reading test match with the findings of cognitive research. For example, STC assessments in large-scale assessments often follow a functional perspective on reading. Reading literacy in this sense encompasses “an individual’s capacity to understand, use, reflect on and engage with written texts, in order to achieve one’s goals, to develop one’s knowledge and potential, and to participate in society” (OECD, 2010, p. 14). Such studies are the Programme for International Student Assessment (PISA; OECD, 1999), the International Adult Literacy Survey (IALS; OECD and Statistics Canada, 1995) or the National Educational Panel Study (NEPS; Blossfeld and Roßbach, 2019).

### Multiple Document Comprehension

Research on understanding multiple documents indicates the need to expand models of STC (e.g., Goldman, 2004; Rouet, 2006; Bråten et al., 2011; Britt and Rouet, 2012). Out of this thought, the documents model framework (Perfetti et al., 1999; Britt and Rouet, 2012) was developed. It suggests that, in addition to these demands of STC, the comprehension of multiple documents requires two additional mental representations: the *integrated situation model* and the *intertext model*, which are the components of the documents model. The former represents the integration of the situation models for each single text, resulting in a global representation of the situation or phenomena described across the texts. This can be challenging when the reader encounters a conflict due to contradictory or incompatible information. In this case, students can either ignore the conflict, reconcile it or accept it as being due to different sources (Stadtler and Bromme, 2014). The intertext model represents information about single sources (e.g., information about the author, purpose or publication medium) as well as its integration across texts. The whole documents model encompasses the linkage between content and source information (e.g., who stated what). Beyond these cognitive representations, Wineburg (1991) found that the strategies of corroboration (comparing information across documents), contextualization (relating information about the documents’ context to prior knowledge), and sourcing (considering information about sources) are important for understanding multiple documents in history, since experts engaged more in such behaviors compared to novices. These strategies have also been identified as important in other domains like science (e.g., Bråten et al., 2011) even if they are occasionally viewed somewhat differently. Due to domain-specific characteristics, benevolence and expertise are important source attributes in science (Stadtler and Bromme, 2014), whereas in history sources are needed particularly in order to contextualize documents (Wineburg, 1991). Although variability has been identified between domains with respect to related practices, there are commonalities, such as engaging in close reading or constructing arguments that explain the logic of claims which apply to nearly every domain (Goldman et al., 2016), which justify considering MDC as a cross-disciplinary competence.

The enhanced demands of MDC are especially important for university students, who face multiple documents regularly when searching for literature in scientific databases or reading texts assigned by their course instructors. Nevertheless, even high school graduates should be able to extract the meaning of multiple documents, analyze and evaluate their content and employ the texts in their own learning process (Common Core State Standards, 2010; Kultusministerkonferenz, 2012). Studies suggest that upper elementary school children are already capable of processing multiple documents (Beker et al., 2019; Florit et al., 2019). Schoor et al. (2020b) found that the MDC of university students correlated statistically significant with their final school exam grades, indicating that high-performing students performed better in an MDC test than low-performing students. Britt and Aglinskas (2002) provided high school and college students with training in handling multiple documents and found that MDC can be modified. Even though disciplines differ in the extent to which they require students to handle multiple documents, there are indications that MDC develops positively during the course of university studies (von der Mühlen et al., 2016). This is in line with Schoor et al. (2020b), who found that master’s students outperform bachelor’s students on MDC-related tasks. Thus, MDC is a competence that seems to develop in school and is needed in order to successfully graduate from university, since university graduates should be able to gather, evaluate and interpret relevant information and derive scientifically sound judgments. Accordingly, relations to final school exam grades and level of university studies can be found (Schoor et al., 2020a, b).

To date, most studies have assessed MDC by means of essays or intertextual inference verification tasks (for an overview see Primor and Katzir, 2018; for an exception see Schoor et al., 2020b). Essay tasks are defined as expressive tasks in which participants have to write a summary based on multiple documents they have read. Since essays are rated with regard to numerous aspects, their scoring is time-consuming. Additionally, they might measure writing skills in addition to MDC (Griffin et al., 2012). Intertextual inference verification tasks, in contrast, are receptive tasks in which participants have to evaluate the veracity of statements by combining information from different texts. Although this method is time-saving and can be objectively scored, it has the drawback of capturing only the integrated situation model and therefore not taking into account the intertext model or the whole documents model. On the other hand, there are studies which investigate sourcing during multiple text comprehension. Some of these focus on source memory (Maier and Richter, 2013; Braasch et al., 2016; Bråten et al., 2016), others on think aloud assessments (Anmarkrud et al., 2014; Barzilai et al., 2015; Strømsø and Bråten, 2014) which are more process-oriented measures of trustworthiness and refer less to sourcing as a retrospective mental model. To overcome the issue of focusing on subcomponents of MDC, Schoor et al. (2020a, b) developed an MDC test addressing all components of the documents model framework (Britt and Rouet, 2012, see section “Multiple Document Comprehension Measure”). However, the authors state that the relation between STC and MDC remains unclear.

### Relation of Multiple Document Comprehension to Single Text Comprehension

To summarize the abovementioned information, MDC exceeds the demands of STC in several ways. It frequently requires readers to compare and integrate information not only within but across documents, which becomes apparent in the integrated situation model and intertext model component of the documents model framework (Britt and Rouet, 2012).

Afflerbach and Cho (2009) showed that the differentiation between three categories of strategy use for traditional (single) texts can also be applied to (more extensive) reading strategies used with multiple documents. These categories are identifying and remembering important information, monitoring, and evaluating. Differences between single-text and multiple-document strategies within these categories are due to the different demands of MDC and STC.

According to Rouet (2006), the demands of MDC differ from the demands of STC in three ways: the relationship between documents, the distinction between texts and situation, and the role of source information. Imagine reading a newspaper article about a battle scene claiming that several people were wounded (Text A). First, multiple documents can complement each other in different ways. To illustrate this, a second text on this issue (Text B) might be complementary, and thus fill in gaps left by the first text, or contradictory, thus representing different aspects of a situation. Secondly, multiple documents emphasize the distinction between a text and the situation described. Two or more texts can either describe different situations or – and this is what is typically referred to as “multiple documents” – describe one situation from different or similar perspectives. Imagine that the second text (Text B) states that no people were wounded. Since you do not have prior knowledge about this event, you consider that each scenario has a 50% chance of being true. Another text on the same issue (Text C) provides support for the point of view presented in Text A, thus claiming that several people were injured. Maybe you now think that this scenario is more likely to be true than the scenario claiming that no people were wounded. This example illustrates how the updating of prior knowledge and beliefs affects the comprehension of multiple documents (Richter and Maier, 2017). Thirdly, source information are especially important when reading multiple documents. The documents model framework assumes that readers experience texts as social entities which are embedded in a specific context (Britt et al., 2012). Readers can evaluate each text by devoting attention to the characteristics of the author(s), genre, publication date, intended audience and so on which in turn helps them build a representation of the situation described in the texts (especially whom to believe). Imagine that Text B is from a trustworthy source (e.g., an eyewitness or governmental organization), while Texts A and C were written by less objective authors, such as a protester or politicians who might benefit from the conflict. This will probably lead you to believe that the scenario with no casualties is the true one. Source information is especially important when the reader detects a conflict between the texts, as was shown by Braasch and Bråten (2017). This is more often the case when reading multiple documents than when reading a single text, since authors of multiple documents generally do not coordinate their work.

To date, studies examining the relation between MDC and STC are scarce. A study by Stadtler et al. (2013) found that reading multiple documents increased awareness and description of conflicts in comparison to reading a single text with the same content. This finding is in line with previous research stating that information arranged as multiple documents results in a better integrated mental representation than reading the same information within a single text (Wiley and Voss, 1996, 1999; Britt and Aglinskas, 2002; Bråten and Strømsø, 2006). For example, Britt and Aglinskas (2002) investigated whether the effectiveness of the Sourcer’s Apprentice (a computer-based environment for teaching sourcing skills with multiple documents) was due to the nature of the environment or to the particular materials. They found that the students who were trained with the Sourcer’s Apprentice showed better sourcing performance and tended to write better connected essays than the students who read a text-book version (single text) of the same training materials. These results are consistent with findings of Wiley and Voss (1999), who showed that writing an argumentative essay results in better information integration when reading multiple documents and not a single-text website. Furthermore, some studies measuring MDC via the intertextual verification task also measured participants’ understanding of single texts via an intratextual inference verification task. In the latter task, participants had to evaluate the veracity of statements combining information from different parts of a single text. The results indicate that these STC measures correlate moderately, but statistically significantly with the MDC measures, with correlations between 0.41 and 0.58 (Strømsø et al., 2008, 2010; Bråten et al., 2009; Strømsø and Bråten, 2009; Bråten and Strømsø, 2010b, 2011; Gil et al., 2010; Hagen et al., 2014). However, there are currently no studies comparing the dimensionality of the constructs STC and MDC.

### The Present Study

**FIGURE 1:**
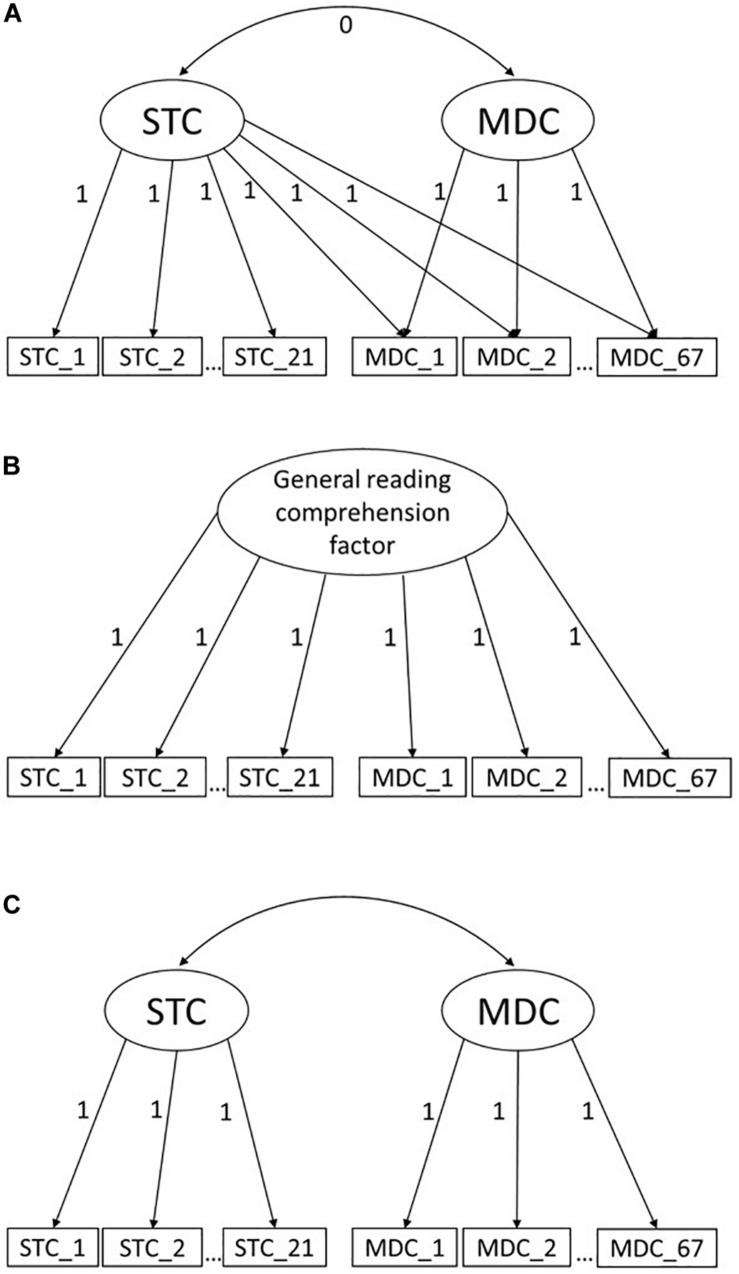
Different approaches for modeling the relation between single text and multiple document comprehension. **(A)** Add-on model, **(B)** Unidimensional model, **(C)** Two-factor model.

Given the recent changes in reading-related demands and parallel changes in theoretical models and assessments of reading comprehension, we were primarily interested in the relation between MDC and STC from an individual differences perspective. Theoretical assumptions, such as the documents model framework (Britt and Rouet, 2012), suggest that the comprehension of multiple documents demands more from readers than STC, which raises the question of how the constructs STC and MDC are related to each other. In the documents model framework by Britt and Rouet (2012), STC is needed in order to complete a multiple document task. Indeed, the STC is inherent in the documents model framework since each document has to be read and understood in order to establish a documents model. Therefore, it can be expected that there is a common underlying factor for both STC and MDC, namely the ability to read and comprehend text. Considering the mentioned changes in reading-related requirements in addition, we hypothesize that not only the ability to read is necessary to solve MDC tasks, but also another ability that is independent of this, such as the ability to integrate multiple perspectives or keep multiple perspectives in mind (in the sense of a working memory-related ability). We call this the add-on hypothesis (for the underlying model see Figure 1A). It suggests that there may be students who are generally very skilled in reading and understanding texts, but who struggle with this additional requirement of MDC tasks. However, two alternative relations between MDC and STC are conceivable as well, as described below.

As a first alternative, STC and MDC might represent the same construct (unidimensionality hypothesis, see Figure 1B for the underlying model). Like our hypothesis, this alternative assumes a common underlying factor for both STC and MDC. In contrast, there is no additional source of inter-individual differences related to solving MDC tasks. Accordingly, students with a low overall ability are expected to score low in both STC and MDC tasks, and students with a high overall ability are expected to score high in both STC and MDC tasks. Differences in performance on STC and MDC tasks are reflected in the item difficulty. This is the approach used by PISA, which is a triennial international study that measures 15-year-olds’ competences in reading, mathematics and science. The new PISA 2018 reading framework (OECD, 2010) expands the reading literacy concept through the use of a few multiple documents tasks. However, in the PISA framework, the multiple document items are not necessarily presented as more difficult than single text items, although single text and multiple document items capture a unidimensional reading construct.

As a second alternative, MDC and STC might be two separable constructs that have different characteristics and refer to distinct single and multiple text reading situations (two-factor hypothesis, see Figure 1C for the underlying model). Accordingly, there will be students who will be more able to comprehend texts in one situation than in the other. However, by expecting a high correlation between these factors, it is recognized that they are related and to some extent based on a number of common underlying abilities (e.g., decoding words and language comprehension). This is the case for competences assessed in different domains within international large-scale assessments. For example, in the PISA studies, reading literacy, mathematical literacy, and scientific literacy are separate constructs but still highly correlated (for PISA 2009 see OECD, 2012). A whole host of factors are probably involved in this covariation, intelligence being one of them (Baumert et al., 2009). Furthermore, STC is also required for mathematical or scientific tasks when formulated in text form (e.g., problem solving tasks, OECD, 1999; Baumert et al., 2009). The two-factor hypothesis differs from the add-on hypothesis in the relation and operationalization of the constructs. In the add-on hypothesis, the additional competence to deal with multiple documents is independent of the competence of reading and comprehending texts. In the two-factor hypothesis the constructs MDC and STC are related since the factors are allowed to correlate. A high correlation between the two factors can be expected.

Based on these considerations, we postulate three competing and mutually exclusive hypotheses:

H1a: MDC is based on STC, but goes beyond reading-specific requirements, since additional cognitive processes are also required (add-on hypothesis).

H1b: MDC and STC represent the same construct (unidimensionality hypothesis).

H1c: MDC and STC represent separable constructs that are highly correlated (two-factor hypothesis).

Furthermore, we were interested in how MDC test scores are related to final school exam grades, the level of university studies and university performance. Since MDC is a competence that can already be observed in upper elementary school children (Beker et al., 2019; Florit et al., 2019) and develops further during the course of university studies (von der Mühlen et al., 2016; Schoor et al., 2020b), we expected that MDC test scores…

… are predicted positively by students’ final school exam grades (H2),

… are predicted more strongly by the final school exam grades among bachelor’s students than among master’s students (H3),

… are higher among master’s students than among bachelor’s students (H4).

To the best of our knowledge, the relation between university performance (indicated by bachelor’s and master’s degree grade point averages) and MDC has not yet been investigated. Nevertheless, since there is evidence that MDC develops positively during the course of university studies (von der Mühlen et al., 2016), and MDC is a necessary component for the successful completion of university, we expected that MDC test scores …

… are predicted positively by prior university performance (H5),

… positively predict expected future university performance (H6).

In light of H1, we were also interested in exploring the relationships specified in H2–H6 conditional on STC. If MDC still relates to these variables even when the shared variance with STC is removed, this will deliver additional evidence that MDC represents a separable construct providing relevant additional information about readers.

## Materials and Methods

### Sample

The original sample consisted of 508 university students from two German universities enrolled in different programs within the humanities and social sciences. In order to prevent bias due to fluency in German, we excluded seven non-native speakers who had been learning German for less than 10 years. The resulting sample of 501 university students still included four non-native speakers who had spoken German for at least 17 years. The participants’ age in the reduced sample spanned from 17 to 42 years (*M* = 22.76, *SD* = 3.77, 78% female). The sample consisted of 53% (*n* = 264) first-semester bachelor’s students and 46% (*n* = 232) master’s students (who had been studying for 1–14 semesters). One percent of the sample (*n* = 5) were teacher education students or students enrolled in old qualification formats like the university diploma (who had been studying for 8–18 semesters). The participants’ final school exam grades (German Abiturnoten) ranged from 1.0 to 3.7 (*M* = 2.12, *SD* = 0.66, *n* = 493). German Abitur grades and final university grades range from 1 (“very good”) to 4 (“sufficient”, pass mark). The bachelor’s degree grade point averages of the master’s students ranged from 1.1 to 2.8 (*M* = 1.83, *SD* = 0.39, *n* = 230). The anticipated master’s degree grade point average of the master’s students ranged from 1.0 to 3.0 (*M* = 1.70, *SD* = 0.34, *n* = 229).

### Design and Procedure

After the students provided their informed consent for participation, the study started with a questionnaire about demographic variables, such as the students’ final school exam grades and level of university studies. Master’s students were also asked for their bachelor’s degree grade point average and anticipated master’s degree grade point average. Afterward, the participants had to complete three blocks, which were presented in randomized order (booklet design). Between these blocks, participants had the chance to take a short break. The blocks consisted of either the MDC test, the STC test or a working memory test, which was not the focus of this study. Each participant completed two out of five units of the MDC test, which were administered in a balanced incomplete block design. They had the opportunity to take a break between units. The entire test session took about two hours. Both tests had a unit structure, as described in the following section.

### Measures

In order to ensure the comparability of the STC and MDC constructs, structurally similar tests were employed in the present study. This means that both tests had a unit structure [a unit is defined by text(s) plus items] and a similar navigation (e.g., participants could return to the texts at any time and texts and items were presented on different pages).

#### Multiple Document Comprehension Measure

The MDC test began with a tutorial that explained the basic functions of the test, such as navigation, how to access source information, note-taking and highlighting. Actually, 74% of the sample paid attention to at least one source. Each MDC unit included two or three texts on the same issue with 11 to 17 items each. The five MDC units consisted of texts from four different domains (2× history, physics, literature, and literature studies) in order to assess MDC as a generic cross-disciplinary competence (for details see Schoor et al., 2020a, b). The texts and items were in German and developed by Schoor et al. (2020b). Most of the texts within a unit were redundant or complementary, only a few contained conflicting information. Conflicts were not fundamental and rather on a detail level, such as information on the age of a protagonist. In order to avoid prior knowledge effects, the text contents were fictitious, except for those in the physics domain. However, since this unit contained texts of a very specific nature, students were not expected to have much prior knowledge about the topic. Two units included an essay task, which had to be completed before the other items could be accessed. However, the essays were not included in the MDC test score, but instead used as a validation criterion. An overview of the text characteristics of the MDC test can be found in Table 1. We report two readability indices. The LIX (“Lesbarkeitsindex”) aims to determine the difficulty of a text by using a formula proposed by Björnsson (1968) and adapted to the German language by Bamberger and Vanecek (1984). It considers the average sentence length of a text and the percentage of words with more than six letters. These are calculated to a total value and compared with experience values of different text genres (high values indicate a more complex test). The Flesch-Reading-Ease (FRE) was originally established by Flesch (1948) and adapted to the German language by Amstad (1978). It results from the average number of syllables per word and the average sentence length. The FRE ranges between 0 and 100 where higher values indicate better comprehensibility (easiness).

**TABLE 1:** Text characteristics of the MDC test.

Unit name	Number of texts	Unit content	Number of items	Number of words^1^	Readability (LIX)^2^	Readability (FRE)^3^	Claimed sources
Universe	3	Texts provide information about the end of the universe from a physics and cosmology perspective	15	455, 464, 448	41.5–45.5	50–55	Newspaper articles
Catalano	2	Biographies on the life of the fictitious mafia boss Catalano	11	644, 584	46.4–49.6	45–52	Online article from a criminological institute; economic newspaper article
2134	3	Texts describe an event in the year 2134: the arrival of aliens on earth	11	491, 434, 381	50.7–54.2	29–43	Internal laboratory report; internal government report; political speech
Nothing	2	Reviews of the fictitious novel ‘Nothing’	13	723, 562	47.1–51.8	43–51	Newspaper articles
Animals	3	Texts talk about different fictitious approaches to interpreting animals in novels	17	629, 1057, 451	51.1–55.0	32–40	Introductory textbook texts

**^1^Number of words per text. ^2^Readability index (LIX) calculated by psychometrica.de (Lenhard and Lenhard, 2014). ^3^Readability index (FRE) calculated by fleschindex.de. The readability indices are measures of text difficulty*.*

**FIGURE 2:**
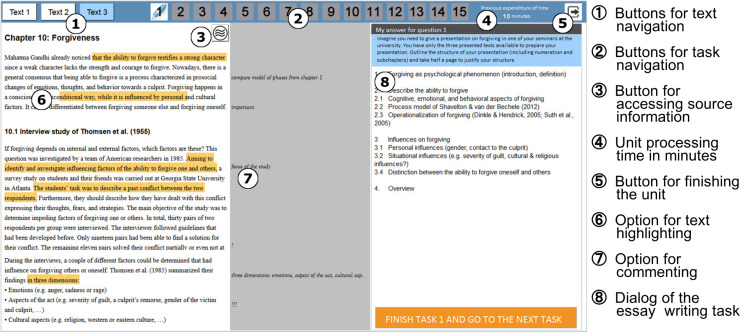
Example of an MDC unit. Source: Hahnel et al. (2019a). Validating process variables of sourcing in an assessment of multiple document comprehension.

Each unit started with an introductory page informing students about the number of texts and items, the time limit and setting a reading goal (e.g., “Please read the texts as if afterward you would have to describe how animals in novels can be interpreted”). The next page displayed the first text of the corresponding unit (for a screenshot of the text see Figure 2). The screen had a top bar with buttons for navigating between texts and items as well as information about the elapsed time and an exit button. During the test session, participants could navigate freely between the texts and items. Each text page included a button that produced a popup dialogue presenting source information about the text. Students could also highlight text passages, write comments in the margins, and receive feedback on their unit processing time and task progress (symbolized by green ticks on the item number buttons). The time restrictions were unit-specific and varied between 27 and 38 min. The units could be exited at any time before the time limit expired by clicking on the exit button. This evoked a popup window with a reminder of unsolved tasks (if any), asking students whether they wanted to exit the unit or return to the tasks. Ten minutes before time ran out, a popup window reminded participants of the already elapsed time and the unit-specific time limit. When the time limit expired, another popup window informed the students that time had run out. They then had to click a button in order to continue with the next task.

The 67 MDC items each measured one out of four cognitive requirements. Items requiring the corroboration of information (19 items) required participants to locate and compare information across different texts and were inspired by Wineburg (1991). The other three item types were constructed with reference to the documents model framework by Britt and Rouet (2012). In order to solve items requiring the integration of information, relevant information had to be identified from the texts and integrated with one another (integrated situation model, 18 items). For items requiring the comparison of sources, text characteristics and source information had to be assessed and compared (intertext model, 16 items). The most complex items were those requiring the comparison of source-content links, since they combine the integrating information and comparing sources requirements (documents model, 14 items). In order to solve items of this type, readers had to build a mental model combining content and source information (who stated what). The MDC items required the consideration of at least two texts in order to identify the correct answer. The items were administered in a single-choice format (1 out of 4) or a verification format (yes/no or true/false). Example items of an excluded unit for each of the cognitive requirements can be found in the Appendix. Schoor et al. (2020a) showed that this MDC test is objective, reliable and valid, and represents a unidimensional construct (rather than a four-dimensional model representing the cognitive requirements or a five-dimensional model representing the unit structure).

Due to technical problems, the MDC data from 8 participants could not be used. For the remaining 493 participants, there were only a small number of missing values due to omitted or not reached items (0.57%). Because of this small amount, missing values were treated as if the respective item had not been administered (Pohl et al., 2014).

#### Single Text Comprehension Measure

The administered STC measure is a computer-based reading comprehension test for university students based on the literacy concept from the National Educational Panel Study (NEPS; Gehrer et al., 2013). The same test was used as an online test for the university student cohort of the NEPS (Rohm et al., 2019). It consists of 21 items spread over five single texts on different topics with three to six items each. The texts represented a range of different text types (i.e., an information text, a commentary text, a literary text, an instructional text and an advertising text). No source information was presented for the texts since some items asked in particular for the source of the text. An exception was the short story unit, in which source information were presented before the text content in order to contextualize the text. See Table 2 for more information on the STC test.

**TABLE 2:** Text characteristics of the STC test.

Unit name	Text characterictics	Number of items	Number of words	Readability (LIX)^1^	Readability (FRE)^2^	Text type
Handicraft	Text conveyed user guidance through work instructions; it is action-oriented and explains an activity step by step	4	238	45.4	51	Instruction text
Journalism	Text takes a particular stance; characterized by an argumentative text structure which is rather complex	5	258	51.2	51	Commenting text
False color photography	Sophisticated text for learning, advanced acquisition of knowledge, and finding detailed information	6	305	57	36	Information text
Law changes	Sophisticated call/claim with a persuasive function; the text language is purpose-oriented	3	250	64.3	22	Advertising text
Short story	Short story with many linguistic means; text with demanding interpretation because of its ambiguity, complexity, compression and openness	3	395	30.3	72	Literary text

**^1^Readability index (LIX) calculated by psychometrica.de (Lenhard and Lenhard, 2014). ^2^Readability index (FRE) calculated by fleschindex.de. The readability indices are measures of text difficulty*.*

The STC test started with a tutorial explaining to students the structure of the test (5 units with one text plus several items each), the total time limit of 28 min for the whole test, and the navigation and item response formats. There was no reading goal presented in the STC test. During the test, students could navigate freely between the text and items within a unit. Highlighting and commenting on text passages was not possible in the STC test. To exit the unit, an arrow button could be clicked any time. Clicking on the arrow button produced a popup window asking students whether they wanted to exit the unit or return to the tasks. When the time limit expired, a popup window informed the students that the time had run out. They had to click a button in order to continue with the next task.

The STC test consisted of items with different cognitive requirements, which had to be answered for each text. Items of Type 1 required students to find detailed information in the text (e.g., “What is xy?”; 2 items). In Type 2 items, text-related conclusions had to be drawn (e.g., “Which assumption about xy can be derived from the text?”; 9 items). The third item type required students to reflect on and assess statements made in the text (10 items). This included the ability to either comprehend the central message of the text, recognize its intention and judge its trustworthiness, or integrate prior knowledge in order to answer the items correctly. A situation model or mental model of the text was required in order to correctly answer Type 3 items, which were mostly items where headings had to be matched to certain paragraphs (matching items) or a new sentence had to be integrated into the text (text enrichment items). These formats are described in the following paragraph. Table 3 shows how the requirements of the STC test correspond to the requirements of the MDC test.

**TABLE 3:** Common requirements of the MDC and STC test.

Requirements MDC test	Corresponding requirement STC test	Common requirement
(1) Corroboration of information across texts: find information in text and compare it across texts.	(1) Finding information in text: find detailed information on sentence level.	Find information.
(2) Integration of information across texts: information has to be combined additively or by means of an inference.	(2) Drawing text-related conclusions: construct local or global coherence. (3a) Reflecting and assessing: comprehend the central idea, integration of background and world knowledge.	Integrate information.
(3) Comparison of sources and source evaluations across texts: judge each single source and compare.	(3b) Reflecting and assessing: recognize purpose and intention of a text, judge credibility.	Judge information with regard to source features.
(4) Comparison of source-content links across texts.	–	–

The items were presented in one of the following four item formats. Most of the items were administered in a single-choice format, where one answer out of four is correct. Another item format comprised decision-making items where statements about the content of the text had to be judged as correct or incorrect (similar to the verification format in the MDC test). In the matching item format, headings had to be selected and assigned to a text section via drag and drop. Examples for these item formats can be found in Gehrer et al. (2012). The fourth item format comprised text enrichment task items. In these items, participants were asked to enrich a text meaningfully with three to four additional sentences. In order to do so, they had to drag a symbol marking a specific sentence to the correct gap within the text (the sentence could be dragged to any gap between two sentences). More information on the last item format can be found in Rohm et al. (2019). All item formats except for the single-choice items consisted of several subitems, which were summarized during data analysis in order to produce partial credit item solutions. Rohm et al. (2019) showed that the STC test represents a unidimensional construct (rather than a three-dimensional model representing the cognitive requirements or a five-dimensional model representing the unit structure).

Due to technical problems, we had to exclude the STC data from 2 participants. The remaining 499 participants omitted 0.36% of the items and did not reach 3.86% of the items. The missing responses were ignored and thus treated if not administered (same approach as for the MDC test). This is the approach used in the NEPS for scaling data from competence tests (Pohl and Carstensen, 2012). The MDC and STC tests were implemented using the CBA ItemBuilder (Roelke, 2012).

#### Background Variables

In addition to the tests we asked the participants about their final school exam grades and their level of university studies (bachelor, master, and others). Master students were also asked about their bachelor’s degree grade point average and their anticipated master’s degree grade point average (assessed with the following question: “With what grade do you expect to complete your master’s degree?”). All of these background variables were self-reports.

### Data Analysis

In order to investigate the three H1 alternatives, confirmatory (multi-dimensional) item response models specifying the dimensionality of MDC and STC were estimated and compared, using the software R version 3.6.0 (R Core Team, 2019) and the R package *TAM* (Robitzsch et al., 2019). MDC and STC were modeled as latent variables, assuming a Rasch model for MDC responses and a partial credit model (Masters, 1982) for STC responses. This resulted in fixing the discriminations across all items in each model to one (see Schoor et al., 2020a, b).

In order to evaluate the hypothesis that MDC is an additional requirement to STC (H1a), we first specified a two-dimensional model with crossloadings and fixed covariance. That is, both STC and MDC items loaded onto one factor called “STC,” since we assumed that STC is required in order to solve MDC test items correctly. The MDC items additionally loaded onto a second factor which accounted for the “additional requirements” needed to solve the MDC items (add-on model, see Figure 1A). The covariance between the STC and MDC factors was fixed to be zero. In order to evaluate the hypothesis that MDC and STC reflect the same construct (H1b), we specified a unidimensional model where both STC items and MDC items loaded onto a joint factor, which we call “general reading comprehension factor” (unidimensional model, see Figure 1B). In order to examine the hypothesis that MDC and STC are two separable constructs (H1c), we specified a two-dimensional model where STC items loaded onto one factor and MDC items loaded onto another factor (two-factor model, see Figure 1C). The covariance between the two factors was freely estimated. Models were compared by using the Akaike information criterion (AIC), the Bayesian Information Criterion (BIC) and the χ^2^-difference test. We tested the χ^2^-difference for nested models, specifically for the unidimensional and the two-dimensional model as well as between the unidimensional and the add-on model.

Latent regression analyses for H2-H6 were conducted in Mplus 8.1 (Muthén and Muthén et al., 1998-2017). In order to account for the missing data structure of the MDC variables (missing by design), the MLR estimator was used, which allows maximum likelihood estimation with robust standard errors. MDC and STC were modeled as latent variables in a format that depended on the results of H1. Relevant predictors were final school exam grades, level of university studies (0 = bachelor, 1 = experienced students, i.e., master’s or diploma program), bachelor’s degree grade point average (only for master’s students) and anticipated master’s degree grade point average (only for master’s students). Two regression models were tested for each predictor: the first estimated the effect of the predictor (e.g., final school exam grades) on MDC and the second added STC as a further predictor. In order to test whether bachelor’s students differed from more experienced students in the relation of their final school exam grades to MDC (H3), a Wald test was performed.

## Results

### Dimensionality of Multiple Document Comprehension and Single Text Comprehension (H1)

Table 4 shows the results of the structural analyses of the STC and MDC test items. Both the AIC and the BIC showed higher values for the add-on model and the unidimensional model than for the two-dimensional model, indicating that the two-dimensional model better fit with the data. Furthermore, the χ^2^-difference test showed that the two-dimensional model was statistically significantly different from the unidimensional model. Therefore, the results support H1c. The latent correlation between the latent factors MDC and STC was *r* = 0.84.

**TABLE 4:** Structural analysis of STC and MDC test items and model comparison with the unidimensional model.

Model	AIC	BIC	*n*_Par_	Δχ ^2^	Δ df	*p*
Add-on model	30615.70	31050.01	103	4.90	1	0.03
Unidimensional model	30618.59	31048.69	102			
Two-factor model	**30574.04**	**31012.57**	104	48.55	2	0.00

**n_*Par*_, number of parameters. Lowest values of the AIC and BIC indicating best fit are in bold.**

### Relations to Final School Exam Grades, Level of University Studies and University Performance (H2–H6)

Below, results are reported regarding the relations between the MDC test and final school exam grades, level of university studies and university performance. For comparative purposes, we also report the impact of the aforementioned variables on the STC test. MDC and STC were modeled as latent variables following the two-dimensional model.

#### Final School Exam Grades (H2)

Final school exam grades statistically significantly predicted STC (β = −0.39, *p* < 0.001) as well as MDC (β = −0.43, *p* < 0.001). This means that a better (lower) final school exam grade was associated with a better MDC test score, supporting H2. When STC was included as predictor in the regression model of MDC on final school exam grades, the impact of final school exam grades on MDC became smaller, but was still statistically significant (β = −0.24, *p* < 0.001). As to be expected, STC also statistically significantly impacted MDC test scores (see Table 5).

**TABLE 5:** Standardized effect sizes of the impact of the level of university studies, final school exam grades and prior university performance and STC on MDC per hypothesis.

		H2: Final school exam grades	H3: Level of university studies and final school exam grades		H4: Level of university studies	H5: Prior university performance
			Bachelor’s students	Master’s students		
Impact on STC	β_Predictor_^1^ (*SE*)	−0.39*** (0.05)	−0.38*** (0.07)	−0.44*** (0.07)	0.23*** (0.05)	−0.28*** (0.08)
Impact on MDC	β_Predictor_^1^ (*SE*)	−0.43*** (0.05)	−0.49*** (0.06)	−0.40*** (0.08)	0.24*** (0.06)	−0.31*** (0.07)
Impact on MDC when including STC as predictor	β_Predictor_^1^ (*SE)*	−0.24*** (0.05)	−0.33*** (0.07)	−0.13 (0.08)	0.11* (0.05)	−0.17* (0.08)
	β_*STC*_ (*SE*)	0.78*** (0.05)	0.73*** (0.06)	0.81*** (0.05)	0.82*** (0.04)	0.75*** (0.06)

*Prior university performance (bachelor’s degree grade point average, H5) could only be examined for the subsample of master’s students. ^1^Predictor varies depending on the hypothesis (H2 and H3: final school exam grades, H4: level of university studies, H5: bachelor’s degree grade point average).* ***p < 0.001, *p < 0.05.

#### Level of University Studies and Final School Exam Grades (H3)

The (negative) relation between the final school exam grades and MDC was higher for bachelor’s than for experienced students (β_BA_ = −0.49, *p*_BA_ < 0.001; β_MA_ = −0.40, *p*_MA_ < 0.001), but the difference was not statistically significant [χ^2^(1) = 0.39, *p* = 0.534]. The opposite was found for STC (β_BA_ = −0.38, *p*_BA_ < 0.001; β_MA_ = −0.44, *p*_MA_ < 0.001), but again the difference was not statistically significant [χ^2^(1) = 1.97, *p* = 0.161]. When STC was included as predictor in the regression model, the impact of final school exam grades on MDC became smaller and was only still statistically significant for bachelor students (β_BA_ = −0.33, *p*_BA_ < 0.001; β_MA_ = −0.13, *p*_MA_ = 0.106). However, the difference was not statistically significant [χ^2^(1) = 2.28, *p* = 0.131]. STC also statistically significantly impacted MDC test scores in both groups (see Table 5).

#### Level of University Studies (H4)

Level of university studies statistically significantly predicted MDC test scores (β = 0.24, *p* < 0.001). The same was true for STC test scores (β = 0.23, *p* < 0.001). The positive β coefficient indicates that more experienced students, such as master’s students, performed better on the MDC test than bachelor’s students. When STC was included as predictor, the impact of the level of university studies diminished, but was still statistically significant on the 5% level (β = 0.11, *p* = 0.030). STC had a statistically significant impact on MDC (see Table 5).

#### Prior University Performance (H5)

University performance could only be examined for the subsample of master’s students. The bachelor’s degree grade point average of master’s students statistically significantly predicted their MDC test scores (β = −0.31, *p* < 0.001) and STC test scores (β = −0.28, *p* < 0.001). When STC was included as predictor, the impact of bachelor’s degree grade point average on MDC diminished, but was still statistically significant on the 5% level (β = −0.17, *p* = 0.025). STC had a statistically significant impact on MDC (see Table 5).

#### Expected Future University Performance (H6)

Multiple document comprehension statistically significantly predicted the students’ anticipated master’s degree grade point average (β = −0.32, *p* < 0.001, see Table 6). This was not true for STC (β = −0.15, *p* = 0.075). The impact of MDC on anticipated master’s degree grade point average was even higher when STC was included as predictor as well (β = −0.49, *p* < 0.001). The shared variance of STC and MDC could not be responsible for this effect, since the effect of MDC on anticipated master’s degree grade point average already existed before including STC. Therefore, the statistically significant relationship is not traceable to STC, but only to MDC, indicating that MDC explains anticipated master’s degree grade point average.

**TABLE 6:** Standardized effect sizes of the impact of STC and MDC on expected future university performance.

		H6: Expected future university performance
Impact of STC on anticipated master’s degree grade point average	β_*STC*_ (*SE*)	−0.15 (0.08)
Impact of MDC on anticipated master’s degree grade point average	β_MDC_ (*SE*)	−0.32*** (0.08)
Impact of MDC on anticipated master’s degree grade point average when including STC as predictor	β_MDC_ (*SE*)	−0.49** (0.18)
	β_*STC*_ (*SE*)	0.23 (0.18)

*Expected future university performance could only be examined for the subsample of master’s students.* ***p < 0.001, **p < 0.01.

## Discussion

The main purpose of this study was to examine the relation between single text and multiple document comprehension, since theoretical assumptions, such as the documents model framework (Britt and Rouet, 2012), suggest that the comprehension of multiple documents demands more from readers than the comprehension of single texts. We further investigated the relation between final school exam grades, the level of university studies and university performance with test scores for each form of text comprehension and explored these variables’ relations to MDC while including STC as predictor in order to shed light on the relationship of MDC and STC.

### Discussion of the Results

With regard to the relation of STC and MDC, confirmatory dimensionality analyses revealed that a model with two separable but correlated factors (i.e., STC and MDC, latent correlation: 0.84) had a better fit compared to an add-on model and a unidimensional model. Accordingly, there is evidence in favor of two highly correlated, but separable constructs (H1c) rather than MDC representing an add-on in terms of cognitive demands (H1a) or a single reading construct (H1b). The latent correlation found between STC and MDC resembles, for instance, the latent correlation between science literacy and reading literacy (*r* = 0.87) or science literacy and mathematics (*r* = 0.89) in PISA 2009 (OECD, 2012), indicating that STC and MDC are indeed separable constructs. This implies that single and multiple text reading situations are different in terms of the cognitive requirements they place on readers. There are students who perform better in single text reading situations than in multiple text reading situations and vice versa. We assume that the high correlation between these factors can be traced back to common underlying abilities, such as the decoding of words and sentences or intelligence. Thus, we cannot tell if MDC items are more difficult than STC items, but they require different – although related – abilities. This supports the view that additional (different) cognitive requirements are needed in order to represent multiple documents compared to single texts (Britt and Rouet, 2012) and is in line with Rouet (2006), who postulated that the demands of single text and multiple document comprehension differ. The results also suggest that the MDC test was successful in focusing on the nature of multiple document comprehension.

Furthermore, we could replicate the relations between the level of university studies (bachelor or master studies), final school exam grades and MDC (H2-H4) found by Schoor et al. (2020b), who used the same MDC test on a different sample. Our results show that final school exam grades statistically significantly predicted MDC (supporting H2), that MDC is not predicted more strongly by final school exam grades among bachelor’s students than among master’s students (rejecting H3), and that MDC is higher for master’s students than for bachelor’s students (supporting H4). Additionally, we added new findings to the existing literature since we found that prior university performance positively predicted MDC test scores (supporting H5) and that expected future university performance in terms of anticipated master’s degree grade point average was predicted by MDC test scores (supporting H6). The same relations were found with STC. The only exception was that STC did not statistically significantly predict anticipated master’s degree grade point average, while MDC did. This indicates that students’ estimation of their expected master’s degree grade point average to some point relied on their MDC and not on their STC, which is reasonable since MDC is particularly important during master’s degree programs. However, a reciprocal relationship of grade point averages and both MDC and STC would also be conceivable.

In addition, we found smaller but still statistically significant relationships between MDC and the analyzed variables when STC was included in the models except for the impact of final school exam grades on MDC for master’s students. This delivers additional evidence that MDC represents a construct that differs from STC, since it provides relevant additional information about readers.

The finding that STC and MDC are highly correlated, yet separable constructs is interesting. It suggests that theoretical models explicitly addressing MDC – like those proposed by many researchers since the late 1990s – are reasonable and necessary. However, it has to be considered that we did not assess situation models for each single text in the MDC test. Although this is true for an explicit assessment, individual situation models were assessed implicitly since at least two texts had to be read and understood in order to answer the MDC items correctly. Our results suggest that MDC and STC are highly related (and not independent) constructs; therefore they support the assumption of Britt et al. (1999) and Perfetti et al. (1999) that in most circumstances situation models are not built for each text, but that the initial situation model is updated during the course of reading. Separate situation models are only created in special circumstances, such as when sources are distinct and elaborated (separate representation model) or when encountering conflicting information which necessitates the creation of an intertext model [tagging of information and corresponding sources, see Britt et al. (1999)]. This view is also consistent with Kintsch who postulates that a network is iteratively created, modified, and updated during the course of comprehension (Kintsch, 1998). Since the documents used in the MDC test are mostly redundant or complementary, it may not have been necessary to build separate situation models.

The strength and novelty of the present study lies in its operationalization of the MDC construct, since the employed test measures the concepts in its pure form. Measuring MDC with a test that covers all components of the documents model framework rather than only the integrated situation model or intertext component is quite novel. However, this implies that the constructs used in the present study differ from recent assessments of reading (literacy) implemented in studies like PISA or the Programme for the International Assessment of Adult Competencies (PIAAC). These large-scale assessments focus on the demands of authentic reading situations, which are conceptualized as a mixture of single text and multiple document comprehension. In the present study, we defined a multiple document task as a task that is not solvable with only one text. This is not necessarily the case in PISA 2018 (OECD, 2019), where multiple documents are viewed as a text characteristic and formats like online forums are also considered to be multiple documents. The approach taken here is thus different from the one taken by the PISA or PIAAC reading assessments.

However, measuring reading comprehension is actually a challenging task. There are different perspectives on reading which will influence how this competence is measured. In the present study we focused on an individual difference perspective by understanding reading as a product. However, there are also other perspectives, such as the cognitive psychological perspective which focusses on the process of reading such as decoding (word level and sentence level) or the educational-psychological perspective which focusses more on fostering reading comprehension. Even large-scale studies measure reading comprehension in different ways. Therefore, the results cannot be generalized to other STC or MDC tests than the ones used in the present study. However, it would be interesting to examine the relations between the MDC test and other STC tests in order to see if the results can be replicated (especially with regard to the challenges associated with measuring reading competence).

Beyond the perspective of reading as a product, computer-based reading assessments can shed light on the behavioral process of reading by means of process (log file) data, that is, how readers proceed when reading single or multiple texts. It would be possible to compare successful with unsuccessful readers and if they differ in the strategies they used or in time they spent on the texts. For example, Heyne et al. (2020) found that instructed highlighting correlated significantly with reading competence. Therefore it might be one of the strategies used by successful readers. Another factor that plays a role in comprehending documents is the readers’ working memory. Research relating working memory and MDC is still in the early stages (e.g., Hahnel et al., 2019b), but shows that MDC is cognitive demanding for university students. Future research should further investigate the revealed commonality between STC and MDC by identifying the common source of variance.

### Limitations

As a first limiting factor, it should be noted that the results of the present study are based on an *ad hoc* sample. Accordingly, the participating university students were not representative for the respective overall student population in the social sciences or humanities, but rather drawn from an easily accessible part of the population. Therefore, the results of the study are not generalizable to other student populations. In this regard, attention should also be paid to the anticipated master’s degree grade point average. Students might tend to overrate future test results since the expected mean grade point average of the master’s degree is descriptively slightly better than the mean of the bachelor’s degree (*M*_Master_ = 1.70; *M_Bachelor_* = 1.83). However, this could also indicate a potential selection bias due to self-selection (e.g., regarding the decision to continue studying) or external selection (e.g., numerus clausus or entrance tests). This would mean that especially students with better (lower) bachelor’s degree point averages decide to study in a masters’ program and that they are more likely to be admitted to these programs.

Secondly, it should be noted that students were provided with fictitious information in the MDC test (except for the unit “Universe”). This was done in order to minimize the impact of prior knowledge and prior beliefs, but goes along with the restriction that the MDC test can only rudimentarily capture what is often needed when dealing with multiple documents in everyday life: setting one’s own preferences aside and processing information that is inconsistent with one’s convictions as well as assessing the credibility of sources. Since the documents of the MDC test do not address critical or ambiguous topics, students were not explicitly informed about the fact of reading fictitious information. Nevertheless, debriefing should be considered in future studies.

A third limiting factor concerns the comparability of the MDC test and the STC test. First of all, an explicit reading goal was only provided in the MDC test, but not in the STC test. Research has shown that reading goals can affect reading decisions, reading processes, and reading outcomes (e.g., Rouet, 2006; McCrudden and Schraw, 2007). Basically, it is difficult to attribute the differences between the tests to the presence of a reading goal since they also differ in other respects, such as the text length, readability, functionalities (notetaking and highlighting) and time feedback. For example, while the MDC test provided per-minute time feedback on the top of the screen and a time limit reminder 10 min before the time expired, the STC test did not provide such feedback. However, the percentage of not reached items in the STC test was rather low, indicating that most participants were able to complete the entire test in time. As a general limitation, there are several confounding variables related to the test specifications (e.g., reading goal, special functionalities, and time feedback) that may have had an effect on the results. Since we cannot exclude these effects, it would be highly desirable to replicate the present results with other tests.

### Conclusion

The present study closes a research gap by analyzing the dimensionality of STC and MDC assessed using tests which are structurally comparable and capture the measured concepts in their pure form. We found first evidence that STC and MDC are separable constructs, indicating that single and multiple text reading situations differ from each other in terms of the requirements they place on readers. However, the high correlation between the constructs indicates that fundamental abilities, such as decoding abilities or reasoning, are needed in both situations. This finding is not only important for the context of university studies, but for reading internet texts in general, where texts from multiple sources are prominent. Therefore, reading online can be seen as a special situation of reading multiple documents, since the use of search engines (Google, Bing, etc.) usually leads to information on a topic from different sources with different perspectives. Our work shows that a lack of the ability to understand and integrate information of such multiple texts cannot be compensated by reading skills (even if they are central), but that skills are necessary which are part of critical online reasoning. The present study also contributes to research on the assessment of MDC, since we could replicate the findings of Schoor et al. (2020a) regarding the relations between MDC and the level of university studies and final school exam grades. Furthermore, we could add results on the relation between university performance and MDC.

In summary, the present study enhances our understanding of the MDC construct and its relation to STC as well as to students’ level of university studies, final school exam grades and university performance. We thereby add empirical evidence to the existing research regarding commonalities and differences between MDC and STC, which is currently mostly of a theoretical nature. The present study also shows that the MDC test developed by Schoor et al. (2020a) is an instrument that validly distinguishes MDC from STC and can therefore serve as a diagnostic instrument for university students.

## Data Availability Statement

The raw data supporting the conclusions of this article will be made available by the authors, without undue reservation, to any qualified researcher. The MDC test is currently available on request. It is planned to make the test and the data of the study available to researchers. The STC test is part of a long-term large-scale assessment in Germany (National Educational Panel Study) and is therefore subject to special protective regulations. Access to the test is possible by submitting an application if there is a well-founded interest in research. The use of the test is possible with a cooperation agreement.

## Ethics Statement

Ethical review and approval was not required for the study on human participants in accordance with the local legislation and institutional requirements. The patients/participants provided their written informed consent to participate in this study.

## Author Contributions

NM: data preparation and analysis, manuscript preparation, and final writing. CS and CH: study design, material development, data collection, and final writing. CA: supervision, review, and final approval. FG: review of the manuscript. UK: technical implementation of the study and review of the manuscript. All authors contributed to the article and approved the submitted version.

## Conflict of Interest

The authors declare that the research was conducted in the absence of any commercial or financial relationships that could be construed as a potential conflict of interest.

## Appendix

Example-Items:

Requirement 1: Corroboration of information across texts

• Do the texts agree with regard to the following issues? (agree/disagree)
  • The question whether forgiving is dependent from culture or not.
  • The role of rage for forgiving.

Requirement 2: Integration of information across texts

• Based on the information of all 3 texts: who is the most probable to forgive another person? (1 out of 4).
  • A very religiously living male Asian with difficulties to decide.
  • A female Asian who is very decisive.
  • An atheist male American without contact to the wrongdoer.
  • A Greek nun with lots of contact to the wrongdoer.

Requirement 3: Comparison of sources and source evaluations

• Are the following statements correct? (yes/no).
  • The authors of the texts probably pursue very similar goals.
  • The texts could have been written independently from each other with the authors not knowing the other texts.

Requirement 4: Comparison of source-content links (several sources)

• Compare the three dimensions of factors influencing forgiveness according to Thompsen et al. with the process model by Shavelton and van den Bechele. Which statement is correct? (1 out of 4).
  • All factors influencing forgiveness in the model by Shavelton and van den Bechele can be classified into the dimensions according to Thompsen et al., but not all dimensions according to Thompsen et al. can be assigned to one or more phases of the model by Shavelton and van den Bechele.
  • All dimensions according to Thompsen et al. can be assigned to one or more phases of the model by Shavelton and van den Bechele but not all factors influencing forgiveness in the model by Shavelton and van den Bechele can be classified into the dimensions according to Thompsen et al.
  • Both all dimensions according to Thompsen et al. can be assigned to one or more phases of the model by Shavelton and van den Bechele, and all factors influencing forgiveness in the model by Shavelton and van den Bechele can be classified into the dimensions according to Thompsen et al.
  • Neither can all dimensions according to Thompsen et al. be assigned to one or more phases of the model by Shavelton and van den Bechele nor can all factors influencing forgiveness in the model by Shavelton and van den Bechele be classified into the dimensions according to Thompsen et al.
